# An investigation of the relationship between sPD-1, sPD-L1 and severe pneumonia patients admitted to ICU and its clinical significance

**DOI:** 10.3389/fmed.2025.1605653

**Published:** 2025-06-25

**Authors:** Zirong Gao, Yanhui Lu, Shanshan Yu, Bao Fu

**Affiliations:** ^1^Department of Critical Care Medicine, The Affiliated Hospital of Zunyi Medical University, Zunyi, China; ^2^Department of Neuro Intensive Care Unit, The Affiliated Jinyang Hospital of Guizhou Medical University, Guiyang, China

**Keywords:** severe pneumonia, 28 day mortality rate, soluble PD1, SPDL1, prognosis

## Abstract

**Background:**

Soluble programmed cell death 1 (sPD-1) and its ligand (sPD-L1) have emerged as potential biomarkers for early identification and risk stratification in patients with severe pneumonia (SP). However, there is a lack of robust laboratory evidence supporting their clinical utility. This study aimed to explore the relationship between sPD-1/sPD-L1 levels and clinical outcomes in SP patients.

**Methods:**

This study included SP patients admitted to the Department of Critical Care Medicine at the Affiliated Hospital of Zunyi Medical University between November 2022 and December 2023. Patients were categorized into survivor and non-survivor groups based on 28-day clinical outcomes. Baseline characteristics and laboratory data were collected upon admission. Serum levels of sPD-1 and sPD-L1 were quantified using enzyme-linked immunosorbent assay. Cox regression analysis was performed to identify prognostic factors, and a nomogram was developed to predict outcomes. The predictive performance of sPD-1, sPD-L1, and their combined indices was evaluated using receiver operating characteristic (ROC) curve analysis.

**Results:**

A total of 125 patients with severe pneumonia (SP) were included in this study. Compared to survivors, non-survivors were older, had more severe disease (as indicated by higher SOFA and APACHE II scores), and exhibited lower body mass index (BMI), hemoglobin levels, lymphocyte counts, CALLY index, and albumin levels. Additionally, non-survivors showed significantly elevated levels of systemic inflammatory markers (NLR, PLR, MLR, CLR, CAR, and SII) and higher serum sPD-1 concentrations. Multivariate Cox regression analysis identified age, SOFA score, and sPD-1 levels as independent risk factors for poor prognosis in SP patients. Restricted cubic spline (RCS) curves revealed a linear relationship between age, SOFA score, and the risk of poor prognosis. A nomogram incorporating age, SOFA score, and sPD-1 levels demonstrated strong predictive performance for 28-day mortality in SP patients, with an area under the curve (AUC) of 0.80. Incorporating sPD-1 measurements significantly improves the prognostic accuracy of both SOFA and APACHE II scores in critically ill patients.

**Conclusion:**

sPD-1 levels were significantly elevated in non-surviving SP patients, suggesting its potential role as a biomarker for disease severity and immune dysregulation. The combination of sPD-1 with other clinical parameters may provide valuable insights into the prognosis and immune status of SP patients.

## Introduction

Severe pneumonia (SP) is characterized by acute respiratory failure, severe hypoxemia, and potential multi-organ dysfunction, often necessitating life support. These clinical manifestations arise from the progressive worsening of inflammation in lung tissues, including the fine bronchioles, alveoli, and interstitium (1, 2). According to the 2021 report on the management of community-acquired pneumonia in intensive care units (ICUs), the mortality rate among SP patients admitted to ICUs remains alarmingly high at approximately 30%. In the United States alone, SP contributes to over 100,000 deaths annually, posing a significant public health burden (3, 4).

Immune dysfunction plays a central role in the pathophysiology of SP-related mortality, involving intricate interactions among various immune cells and their molecular regulators (5, 6). Early monitoring and assessment of immune function, coupled with timely diagnosis and risk stratification, have been shown to improve clinical outcomes in SP patients (7, 8). Despite the identification of over 200 biomarkers over the past two decades, only a limited number, such as C-reactive protein (CRP) and procalcitonin (PCT), have demonstrated clinical utility. However, these biomarkers are often limited by low specificity and sensitivity (9). Consequently, there is an urgent need to identify novel biomarkers—or combinations thereof—that can enhance the diagnosis, risk stratification, and therapeutic management of SP.

Programmed cell death 1 (PD-1), a transmembrane receptor belonging to the immunoglobulin (Ig) superfamily, is predominantly expressed on the surface of activated T cells. Its major ligand, PD-L1, plays a critical role as a co-inhibitory molecule in both innate and adaptive immune responses. PD-1/PD-L1 signaling has been shown to be pivotal in regulating immune dysfunction, particularly in conditions such as sepsis (10), the soluble forms of these molecules, sPD-1 and sPD-L1, have emerged as rapid immune biomarkers and have demonstrated utility in the diagnosis and risk stratification of various critical illnesses similar to severe pneumonia (SP) (11–14). However, their specific role in SP remains poorly understood.

This study aimed to investigate the relationship between serum concentrations of sPD-1 and sPD-L1 and disease severity in SP patients. Additionally, we sought to evaluate the prognostic value of sPD-1/sPD-L1 in predicting clinical outcomes, with the goal of providing insights that could enhance the diagnosis, treatment, and prognostic assessment of SP in clinical practice.

## Methods and material

### Study population

This prospective cohort study enrolled 125 SP patients (>18 years) admitted to the Intensive Care Unit of Zunyi Medical University between November 2022 and December 2023. Diagnostic criteria followed the 2015 Chinese Guidelines for Emergency Management of Community-Acquired Pneumonia for severe community-acquired pneumonia (CAP). Exclusion criteria included: (1) Pregnancy or lactation (2) Sepsis from non-pulmonary etiologies (3) Comorbidities affecting biomarker kinetics: Hepatic dysfunction (Child-Pugh class C), Advanced renal disease (CKD stage ≥4 or AKI stage 3), Immunodeficiency disorders, Malignancy, and Current immunosuppressive therapy. Serum samples were collected after obtaining written informed consent from patients or their legal guardians. Serum levels of soluble PD-1 (sPD-1) and PD-L1 (sPD-L1) were quantified using enzyme-linked immunosorbent assay (ELISA). Comprehensive clinical parameters were concurrently recorded for all SP patients.

### Data collection

During hospitalization, baseline demographic and clinical data were collected, including age, sex, underlying disease, comorbidity with shock, days in ICU, 28-day mortality, and routine laboratory markers (white blood cell count, neutrophil count, lymphocyte count, monocyte count, hemoglobin, C-reactive protein, procalcitonin, bilirubin, platelet count, D2 polymerase, albumin, and creatinine) and indicators of disease severity (APACHE II and SOFA scores).

### Blood sample

2 mL of peripheral blood were collected within 24 h of ICU admission; the samples were allowed to stand for 30 min and centrifuged at 1,000 g for 15 min at 4°C, and the supernatant was frozen at-80°C for subsequent analysis.

### Serum sPD-1/sPD-1 L measurement

The sPD-1/sPD-1 L levels were determined using an enzyme-linked immunosorbent assay kit (Shanghai Thrive Color Biotechnology Co., Ltd.) in a multifunctional enzyme labeling instrument (Shanghai Meigu Molecular Instrument Co., Ltd.), and each sample was analyzed in duplicate. The testing procedure was performed according to the manufacturer’s instructions.

### Statistical analysis

SPSS 29.0 software was applied for statistical analysis. All data were tested for normality, and normally distributed measures were expressed as `X ± S, and skewed distributions were expressed as M (*P*25, *P*75). A t-test was used for intergroup comparisons for those with normal distribution, and the chi-square test was used for quantitative value comparisons between two groups for those with chi-square or skewed distribution, and the Kruskal-Wallis test was used for quantitative value comparisons between multiple groups. Correlation analysis was performed using Pearson (normal distribution) or Spearman (skewed distribution). Logistic regression analysis was performed to identify risk factors associated with 28-day mortality. The predictive power of the biomarkers was determined by analyzing the subjects’ work characteristics (ROC) curves and calculating the area under the curve (AUC). *p* < 0.05 indicates a statistically significant difference. Finally, to validate the efficacy of our nomogram, the entire cohort was randomly divided into testing cohorts with distinct clinical characteristics using R’ s built-in sample function, and the validity of our risk prediction model was assessed across both cohorts.

## Result

### Patient characteristics

A total of 125 patients with severe pneumonia (SP) were enrolled in this study, including 84 survivors and 41 non-survivors. The clinical and demographic characteristics, laboratory findings, and prognostic data are summarized in Table 1. Compared to survivors, non-survivors were significantly older, had a lower body mass index (BMI), and exhibited higher Acute Physiology and Chronic Health Evaluation II (APACHE II) and Sequential Organ Failure Assessment (SOFA) scores (*p* < 0.01).

**Table 1:** Patient characteristics.

Variable	Total (*n* = 125)	Survival (*n* = 84)	Non-survival (*n* = 41)	*p*-value
Age	71.00 (57.00,76.00)	66.5.00 (55.75,75.00)	75.00 (67.00,79.00)	<0.01
BMI	24.10 (21.60,26.70)	24.50 (22.38,27.15)	22.80 (20.80,24.30)	<0.01
Gender (*n*, %)				0.540
Man	76 (60.08)	49 (58.33)	27 (65.85)	
Woman	49 (39.92)	35 (41.67)	14 (34.15)	
Complication (*n*, %)				
Hypertension	16 (12.80)	9 (10.71)	7 (17.07)	0.475
CVD	15 (12.00)	7 (8.33)	8 (19.51)	0.130
Diabetes	18 (14.40)	11 (13.10)	7 (17.07)	0.965
COPD	12 (9.60)	6 (7.14)	6 (14.63)	0.312
Shock	84 (67.20)	51 (60.71)	33 (80.49)	0.156
Severity score				
Apache II	24.00 (18.00,28.00)	22.00 (18.00,26.00)	26 (23.00,29.00)	<0.01
SOFA	11.00 (9.00,14.00)	9.00 (9.75,13.00)	13.00 (11.00,15.00)	<0.01
Laboratory examination				
CPR (mg/L)	132.41 (96.54,165.98)	128.94 (88.68,162.89)	149.04 (117.33,172.78)	0.060
PCT (ng/mL)	14.33 (9.21,22.78)	12.49 (7.96,22.17)	16.48 (11.18,24.65)	0.050
D-2 polymer (ug/mL)	4.01 (1.68,6.68)	3.97 (1.56,11.40)	4.05 (1.78,5.45)	0.410
Hemoglobin (g/L)	114.00 (102.00,122.00)	114.00 (104.75,125.00)	108.00 (101.00,119.00)	0.140
WBC	12.34 (8.50,16.34)	12.40 (9.00,15.34)	12.34 (7.58,17.67)	0.840
Neutrophil	9.91 (6.96,13.93)	9.24 (7.09,12.62)	10.23 (6.76,15.55)	0.650
Lymphocyte	0.89 (0.57,1.34)	1.01 (0.60,1.59)	0.75 (0.42,0.99)	<0.01
Monocyte	0.89 (0.50,1.23)	0.90 (0.50,1.22)	0.89 (0.52,1.25)	0.560
PLT	149.00 (98.00,207.00)	149.00 (94.75,192.75)	156.00 (107.00,212.00)	0.910
Albumin (g/L)	35.10 (31.20,41.20)	36.40 (32.28,48.83)	34.50 (32.20,36.70)	0.040
Creatinine (umol/L)	134.00 (81.00,194.00)	125.00 (74.50,184.00)	162.00 (86.00,241.00)	0.070
Bilirubin (umol/L)	19.30 (12.30,50.80)	20.65 (11.40,51.05)	16.80 (12.30,50.10)	0.960
Combination inflammatory indicators				
NLR	12.34 (6.29,22.20)	10.41 (5.44,18.82)	17.37 (9.86,23.57)	<0.01
MLR	1.00 (0.53,1.51)	0.80 (0.44,1.26)	1.34 (0.79,2.06)	<0.01
PLR	136.58 (73.28,244.50)	143.48 (77.36,242.42)	220.39 (123.78,371.05)	<0.01
CLR	135.00 (84.16,261.40)	123.18 (72.88,221.85)	179.98 (133.07,314.50)	<0.01
SII	1611.11 (899.00,3021.93)	1240.19 (723.71,2770.98)	2581.81 (1315.74,3381.40)	<0.01
CAR	3.53 (2.71,4.79)	3.30 (2.58,4.25)	4.15 (3.44,5.32)	<0.01
CALLY	26.80 (13.07,44.38)	32.56 (14.57,52.99)	18.95 (11.07,26.69)	<0.01
Programmed death receptor 1				
sPD-1	182.79 (133.17,231.62)	171.44 (126.96,220.49)	218.60 (171.26,283.03)	<0.01
sPD-L1	19.29 (15.61,28.81)	18.73 (16.69,27.36)	20.61 (15.23,35.91)	0.450

Apache II, Acute Physiology And Chronic Health Evaluation II; SOFA, Sequential Organ Failuer Assessmen; NLR, Neutrophil–Lymphocyte Ratio; MLR, Monocyte-Lymphocyte Ratio; PLR, Platelet–Lymphocyte Ratio; CLR, C-reactive protein-Lymphocyte Ratio; SII, Systemic Immune Inflammation Index; CAR, C-reactive protein-Albumin Ratio; CALLY, CRP-Albumin-Lymphocyte Index.

Laboratory analyses revealed that non-survivors had significantly lower hemoglobin levels, albumin levels, and lymphocyte counts (*p* < 0.01). Among the combined inflammatory indices, non-survivors demonstrated elevated neutrophil-to-lymphocyte ratio (NLR), monocyte-to-lymphocyte ratio (MLR), platelet-to-lymphocyte ratio (PLR), C-reactive protein-to-lymphocyte ratio (CLR), systemic immune-inflammatory index (SII), and C-reactive protein-to-albumin ratio (CAR) (*p* < 0.01). In contrast, the C-reactive-albumin-lymphocyte Index (CALLY index) was significantly lower in non-survivors (*p* < 0.01). Additionally, non-survivors exhibited significantly higher serum levels of soluble programmed cell death 1 (sPD-1) (*p* < 0.01). However, no significant difference was observed in sPD-L1 levels between the two groups. Consequently, subsequent analyses focused primarily on sPD-1.

### Correlation between serum sPD-1 and serum combined inflammatory markers and severity scores

For SP patients, our results showed that serum sPD-1 levels were positively correlated with APACHE II (*R* = 0.346, *p* < 0.01), SOFA (*R* = 0.421, *p* < 0.01), and MLR (*R* = 0.238, *p* < 0.01), and showed a negative correlation with CALLY (*R* = −0.184, *p* = 0.040). There was no correlation with CAR (*R* = 0.050, *p* > 0.05), CLR (*R* = 0.176, *p* > 0.05), NLR (*R* = 0.150, *p* > 0.05), SII (R = 0.138, *p* > 0.05), and PLR (*R* = 0.113, *p* > 0.05) (Table 2).

**Table 2:** Correlation between traditional clinical indicators and serum sPD-1.

Variable	*r*	*p*
ApacheII	0.346	<0.01
SOFA	0.421	<0.01
PCT	0.044	0.625
NLR	0.150	0.093
MLR	0.238	<0.01
PLR	0.113	0.210
CLR	0.176	0.050
SII	0.128	0.159
CAR	0.050	0.580
CALLY	−0.184	0.040

Apache II, Acute Physiology and Chronic Health Evaluation II; SOFA, Sequential Organ Failuer Assessmen; PCT, Procalcitonin; NLR, Neutrophil–Lymphocyte Ratio; MLR, Monocyte-Lymphocyte Ratio; PLR, Platelet–Lymphocyte Ratio; CLR, C-reactive protein-Lymphocyte Ratio; SII, Systemic Immune Inflammation Index; CAR, C-reactive protein-Albumin Ratio; CALLY, CRP-Albumin-Lymphocyte Index.

### Factors associated with 28 days mortality rate in SP patients

**Figure 1:**
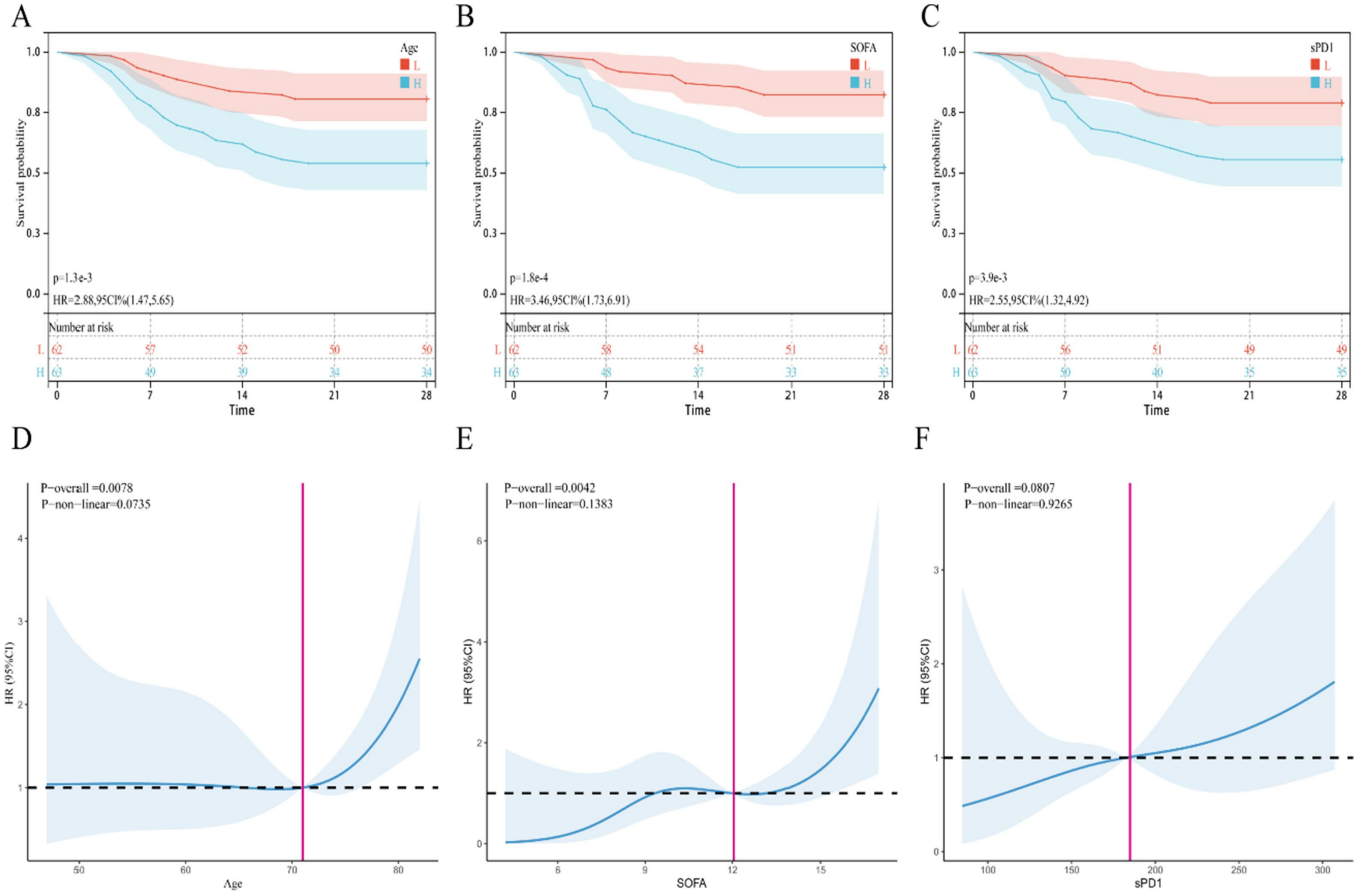
KM survival curve and restrictive cubic spline (RCS) curve. **(A–C)** KM survival curve, **(A)** Age, **(B)** SOFA, **(C)** sPD1; **(D–F)** RCS curve, **(D)** Age, **(E)** SOFA, **(F)** sPD1.

To identify risk factors for death in SP patients, we included statistically significant variables and possible outcome risk factors in baseline comparisons and used multifactorial COX regression analysis to identify factors affecting death in SP patients. Our results showed (Table 3) that after adjusting for age, BMI, APACHE II, SOFA, albumin, lymphocyte count, CRP, NLR, MLR, CLR, and CALLY, the independent risk factor for death in patients with SP was increased age (OR = 1.040, 95% CI: 1.003–1.079, *p* = 0.032), elevated sPD-1 levels (OR = 1.006, 95% CI: 1.001–1.011, *p* = 0.015), and SOFA score (OR = 1.208, 95% CI: 1.037–1.407, *p* = 0.015). Similarly, the KM survival curves further confirmed the association of the above independent risk factors with 28-day mortality in SP patients (Figures 1A–C). Meanwhile, the RCS curves showed (Figures 1C–E), that age and SOFA score were positively and linearly associated with 28-day mortality in SP patients, whereas sPD-1 did not exhibit a linear or nonlinear relationship.

**Table 3:** Univariate and multivariate regression analysis of variables for patient.

Variable	Univariable analysis				Multivariable analysis			
	HR	Lower	Upper	*p* value	HR	Lower	Upper	*p* value
Age	1.056	1.023	1.090	<0.01	1.040	1.003	1.079	0.032
BMI	0.883	0.800	0.973	0.012	0.936	0.845	1.038	0.212
apacheII	1.105	1.053	1.159	<0.01	0.992	0.912	1.079	0.857
SOFA	1.331	1.195	1.482	<0.01	1.208	1.037	1.407	0.015
PCT	1.020	0.999	1.041	0.061	1.005	0.977	1.034	0.717
Lymphocyte	0.448	0.248	0.809	<0.01	0.631	0.196	2.034	0.441
NLR	1.023	1.004	1.041	0.015	1.000	0.968	1.033	1.000
MLR	1.508	1.179	1.929	<0.01	0.852	0.600	1.208	0.368
PLR	1.000	1.000	1.001	0.371				
CLR	1.002	1.001	1.003	<0.01	0.999	0.996	1.002	0.454
SII	1.000	1.000	1.000	0.281				
Albumin	0.943	0.896	0.993	0.027	0.947	0.886	1.011	0.104
CAR	1.425	1.128	1.800	<0.01	1.164	0.760	1.781	0.485
CALLY	0.977	0.961	0.993	<0.01	1.000	0.972	1.028	0.980
sPD1	1.009	1.005	1.013	<0.01	1.006	1.001	1.011	0.015

Apache II, Acute Physiology and Chronic Health Evaluation II; SOFA, Sequential Organ Failuer Assessmen; PCT, Procalcitonin; NLR, Neutrophil–Lymphocyte Ratio; MLR, Monocyte-Lymphocyte Ratio; PLR, Platelet–Lymphocyte Ratio; CLR, C-reactive protein-Lymphocyte Ratio; SII, Systemic Immune Inflammation Index; CAR, C-reactive protein-Albumin Ratio; CALLY, CRP-Albumin-Lymphocyte Index.

### Nomogram for predicting 28-day mortality in patients with SP

**Figure 2:**
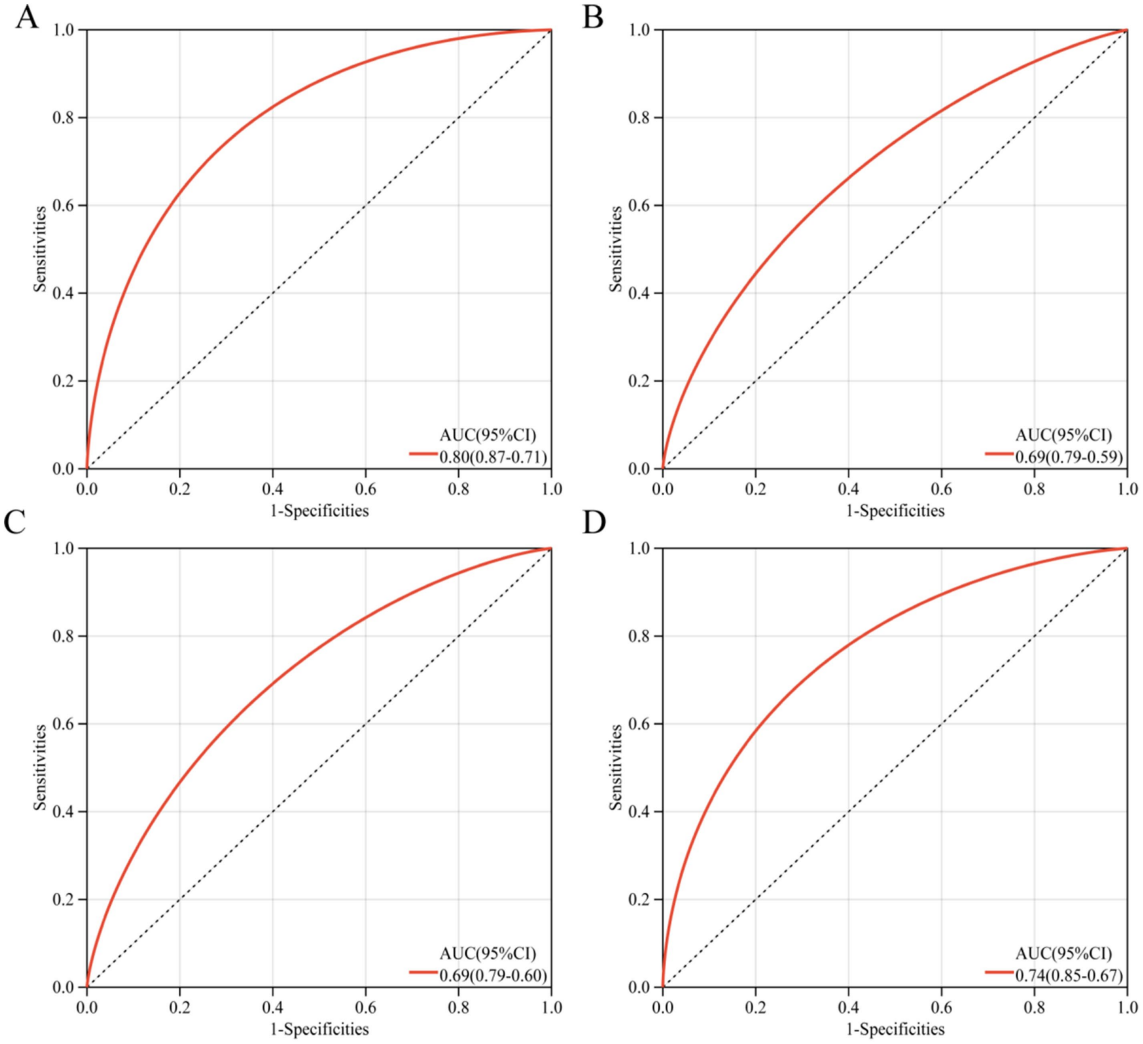
ROC curve. **(A)** nomogram, **(B)** Age, **(C)** sPD1, **(D)** SOFA.

To accurately and early identify SP patients who may die within 28 days, we constructed a column-line diagram (Supplementary Figure S1) based on appealing three (age, SOFA, sPD-1) independent risk factors and assessed its screening effect by ROC curves. The ROC curves showed (Figures 2A–D) that the screening effect of this column-line diagram for 28-day death in SP patients (AUC = 0.80) was greater than age alone (AUC = 0.69), sPD-1 (AUC = 0.69), and SOFA (AUC = 0.74). Furthermore, the risk prediction model consistently demonstrated high predictive accuracy in both validation cohorts (Cohort 1: AUC = 0.80; Cohort 2: AUC = 0.79) (Supplementary Figures S2A,B), further validating the robustness of our findings.

### Incremental effect of sPD1

We assessed the impact of adding sPD1 to existing scoring models (Age, APACHE II, SOFA) on predicting 28-day ICU mortality using area under the curve (AUC) analysis. As shown in Supplementary Figures S3A–C, the inclusion of sPD1 consistently enhanced predictive accuracy across all models: Age (AUC 0.692 → 0.748), APACHE II (0.699 → 0.738), and SOFA (0.738 → 0.776).

## Discussion

Despite significant advancements in recent years, SP remains a life-threatening condition with a poor prognosis, posing substantial challenges in clinical practice (3, 4). The dysregulated immune response associated with SP underscores the need for a deeper understanding of its underlying immune mechanisms and the identification of reliable biomarkers. Such insights could enhance clinical management and ultimately improve patient outcomes (5–8). A critical challenge in managing SP is the lack of a clearly defined transition point from the hyperinflammatory phase to the immunosuppressive phase, making it essential to monitor complex biomarkers that can capture this dynamic shift (15). However, in this study, although we observed that SP patients exhibited higher levels of combined inflammatory indices (NLR, PLR, MLR, CLR, CAR, SII) and serum sPD-1, multifactorial COX regression analysis showed that only sPD-1 could serve as an independent risk factor for SP patients.

PD-1 is an immune checkpoint receptor that plays a critical role in regulating T-cell proliferation and function. Upon binding to its ligand, PD-L1, PD-1 induces immunosuppression by inhibiting T-cell activation, suppressing the release of pro-inflammatory cytokines, and promoting T-cell apoptosis (11, 16, 17). Consequently, the soluble forms of these molecules, sPD-1 and sPD-L1, are increasingly recognized as biomarkers reflecting the immune status of the host (18), this has sparked growing interest in their potential to monitor disease progression in critically ill patients. For instance, a recent study demonstrated that sPD-1 levels are elevated in patients with acute respiratory distress syndrome (ARDS) and may serve as a risk factor for disease progression (15). Similarly, multiple studies have reported elevated sPD-1 levels in the peripheral blood of sepsis patients, suggesting its utility for risk stratification in this population (12, 13, 18). Additionally, research has shown that sPD-1 and sPD-L1 levels are increased in patients with pancreatitis, with higher sPD-1 levels correlating with an elevated risk of pancreatic infection complications (14). These findings collectively highlight the potential of sPD-1 as a diagnostic and risk stratification tool in SP patients, underscoring its feasibility for clinical application in this context.

In our research, we evaluated the predictive ability of sPD-1 for 28-day mortality in severe pneumonia (SP) patients. The receiver operating characteristic (ROC) curve analysis revealed that sPD-1 demonstrated moderate predictive performance (AUC = 0.69). However, relying on a single biomarker often has limitations, including lower sensitivity and specificity, and may not comprehensively reflect a patient clinical status (9, 19). To address these limitations, recent studies have advocated for the use of combined biomarker panels to improve early risk assessment and quantify mortality risk more accurately (19–21). In line with this approach, our study integrated three independent risk factors—age, SOFA score, and sPD-1 levels—to develop a nomogram with enhanced predictive performance (AUC = 0.80). This model provides a more robust tool for quantifying the risk of death in individual SP patients. Furthermore, our findings demonstrate that incorporating sPD1 significantly enhances the predictive accuracy of established risk scores (SOFA and APACHE II) for 28-day ICU mortality in SP patients, underscoring the clinical utility of sPD1 in SP management.

However, the underlying mechanisms linking sPD1 to poor prognosis in severe pneumonia patients remain unclear, though several plausible explanations have been proposed. Previous studies have demonstrated that sepsis can progress to immunosuppression, increasing the risk of secondary infections and poor clinical outcomes (22, 23). Elevated levels of sPD-1 are associated with excessive immune activation. Research suggests that serum sPD-1 may enhance T-cell responses by inhibiting the PD-1/PD-L1 signaling pathway (24, 25). However, persistently high sPD-1 levels may act analogously to PD-1/PD-L1-blocking antibodies, triggering abnormal T-cell activation and proliferation (24, 25). This dysregulated immune response drives hyperinflammation during early sepsis, leading to inflammatory tissue and organ damage (26). Concurrently, lymphocyte depletion shifts the hyperinflammatory state toward immunosuppression, accelerating sepsis progression from immune activation to immune paralysis and ultimately worsening prognosis (22, 23, 26). These findings suggest that markedly elevated sPD-1 levels may reflect more severe immune dysfunction in patients.

In contrast, sPD-L1 binds to PD-1 on T cells, mimicking the immune checkpoint function of membrane-bound PD-L1 to suppress T-cell activation and cytokine production, thereby preventing systemic immune overactivation. Elevated sPD-L1 levels typically indicate an immunosuppressive state, which is strongly linked to poor outcomes in sepsis (22–25). Paradoxically, our data did not identify elevated sPD-L1 as a mortality predictor in severe pneumonia patients. This discrepancy may stem from several factors. First, although sepsis involves coexisting immune activation and suppression, the early disease phase is predominantly hyperinflammatory (22, 23, 26). Thus, SPD-L1 levels may remain low during this stage. Notably, all analyzed serum samples were collected on ICU admission, which may not represent the optimal timeframe for detecting sPD-L1. These observations highlight the need for longitudinal monitoring to evaluate the clinical relevance of serum sPD-1 and sPD-L1 dynamics in guiding severe pneumonia management.

The SOFA score is a key component of the diagnostic criteria for sepsis. Previous studies have demonstrated its utility as an early prognostic marker for predicting 28-day mortality in sepsis patients (27, 28). However, recent research has raised concerns about the limitations of the original SOFA score, particularly in patients with comorbid conditions such as chronic obstructive pulmonary disease (COPD), chronic kidney disease, and malignancies. Despite these limitations, our study found that the SOFA score remains an independent risk factor for mortality in patients with SP, as confirmed by multivariate Cox regression analysis. This underscores the continued relevance of the SOFA score as a valuable tool for risk stratification in SP patients.

This study has several limitations that should be acknowledged. First, as a single-center, prospective observational study, the sample size was relatively small, and all participants were recruited from the ICU, which may introduce selection bias. Second, although elevated serum levels of sPD-1 were observed in non-surviving SP patients, this study only measured sPD-1 and sPD-L1 levels at the time of enrollment. Continuous monitoring was not performed, leaving the potential clinical value of dynamic changes in these biomarkers—such as their ability to track disease progression or predict prognosis—unexplored. Third, while the risk prediction model developed in this study demonstrates strong predictive performance, the absence of external validation means we cannot rule out potential overfitting. Future work should therefore prioritize prospective validation in larger, independent cohorts. Additionally, our study did not account for the influence of causative pathogen type (e.g., bacterial vs. viral) on sPD1 levels. Given that pathogens differentially modulate immune responses, future studies should incorporate larger cohorts and subgroup analyses to clarify their potential impact on clinical outcomes. Finally, while this study focused on the expression levels of serum sPD-1 and sPD-L1 in SP patients, it did not investigate the mechanisms by which these biomarkers influence immune function in SP. Future research, including animal models, is needed to elucidate the pathophysiological relationships between these markers and immune dysregulation in SP.

## Conclusion

In summary, serum levels of sPD-1 were significantly elevated in non-surviving SP patients and showed strong correlations with disease severity scores, including the APACHE II scores and SOFA scores, as well as with various combined inflammatory markers. These findings suggest that sPD-1 and its associated biomarker panels can serve as valuable tools for assessing disease severity and immune status in SP patients. However, further exploration and larger-scale clinical studies are needed to fully elucidate the specific clinical utility and prognostic value of sPD-1 in this patient population.

## Data Availability

The original contributions presented in the study are included in the article/Supplementary material, further inquiries can be directed to the corresponding authors.
