# Supplementary material for: An investigation of the relationship between sPD-1, sPD-L1 and severe pneumonia patients admitted to ICU and its clinical significance
Source: Front Med (Lausanne). 2025 Jun 25;12:1605653. doi: 10.3389/fmed.2025.1605653 (PMC12237637; doi:10.3389/fmed.2025.1605653)
Supplement: Supplementary file 1 [file Supplementary_file_1.docx]

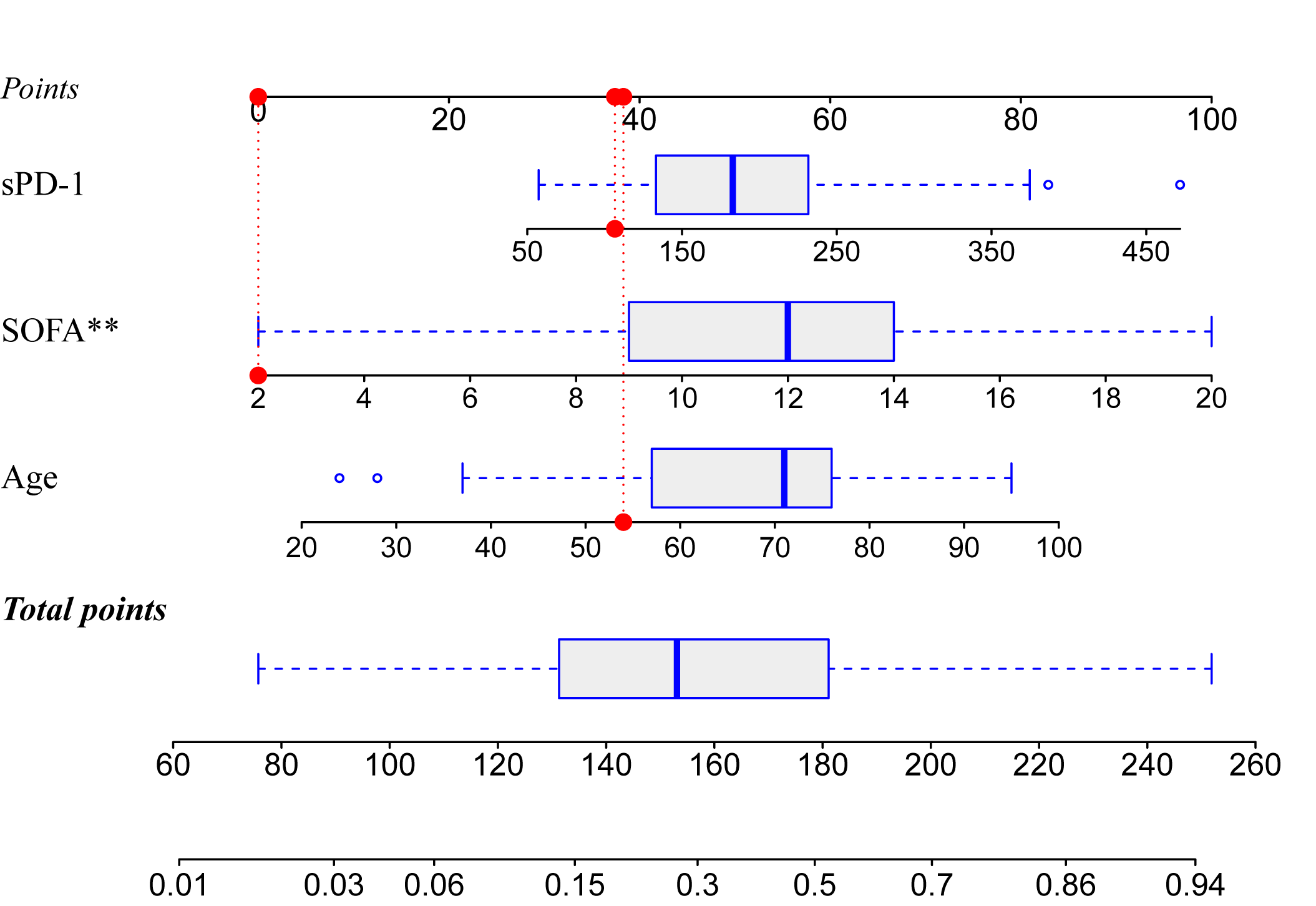


Fig S1: nomogram


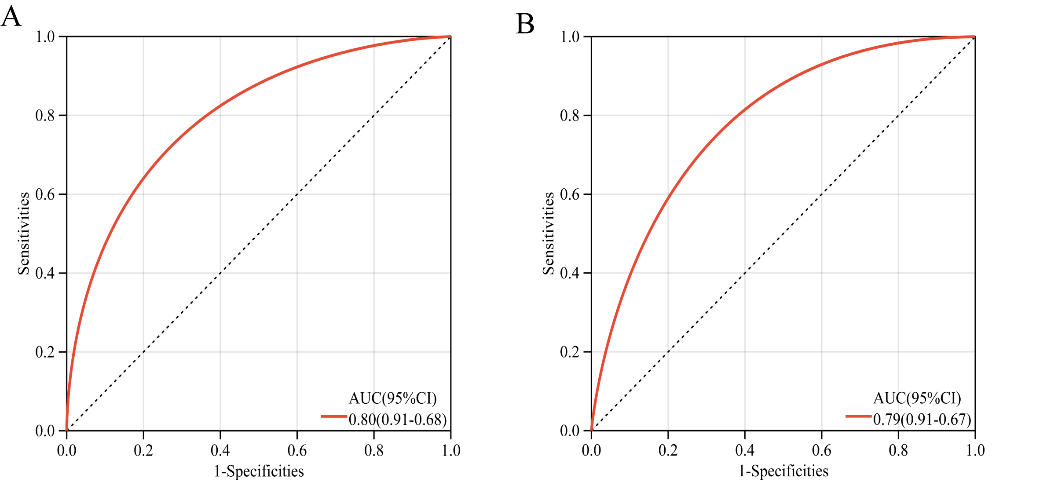


Fig S2: External validation of risk prediction models

Note: A: Cohort 1; B: Cohort 2


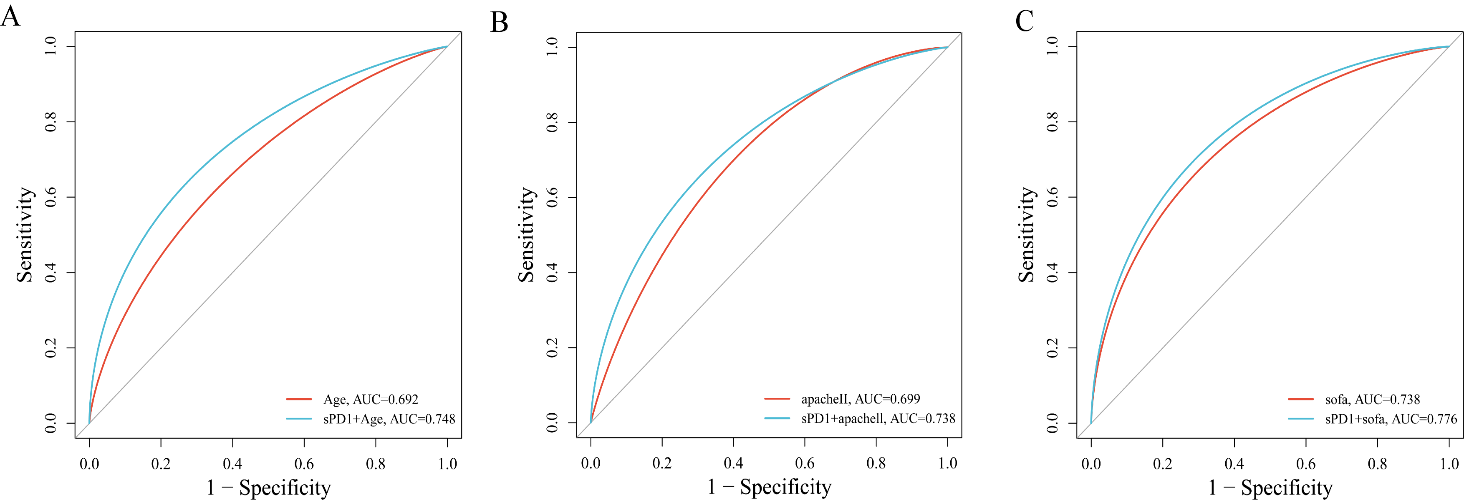


Fig S3: incremental effect of sPD1

Note: A: Joint age; B: Joint APACHE II; C Joint SOFA
